# Hydrogels of Poly(2-hydroxyethyl methacrylate) and Poly(N,N-dimethylacrylamide) Interpenetrating Polymer Networks as Dermal Delivery Systems for Dexamethasone

**DOI:** 10.3390/pharmaceutics17010062

**Published:** 2025-01-05

**Authors:** Marin Simeonov, Bistra Kostova, Rositsa Mihaylova, Elena Vassileva

**Affiliations:** 1 Laboratory on Structure and Properties of Polymers, Faculty of Chemistry and Pharmacy, University of Sofia, 1, J. Bourchier Blvd., 1164 Sofia, Bulgaria; evassileva@chem.uni-sofia.bg; 2 Department of Pharmaceutical Technology and Biopharmaceutics, Faculty of Pharmacy, Medical University of Sofia, 2, Dunav Str., 1000 Sofia, Bulgaria; bkostova@pharmfac.mu-sofia.bg; 3 Department of Pharmacology, Pharmacotherapy and Toxicology, Faculty of Pharmacy, Medical University of Sofia, 2, Dunav Str., 1000 Sofia, Bulgaria; rmihaylova@pharmfac.mu-sofia.bg

**Keywords:** interpenetrating polymer networks, hydrogels, dexamethasone, drug delivery, dermal application, non-cytotoxic, HUT-78, CCL-1

## Abstract

**Background/Objectives**: This study is an attempt to reveal the potential of two types of interpenetrating polymer network (IPN) hydrogels based on poly(2-hydroxyethyl methacrylate) (PHEMA) and poly(N,N-dimethylacrylamide) (PDMAM). These IPNs were evaluated for their potential for dermal delivery of the hydrophobic drug dexamethasone (DEX). **Methods**: The two types of IPNs were analyzed for their rheological behavior, swelling characteristics, and drug-loading capacity with DEX. Drug release profiles were studied in Franz diffusion cells in PBS media. Finally, the cytotoxicity of the PHEMA/PDMAM-based IPNs was studied against T-cell lymphoma cells (HUT-78) and a normal murine fibroblast cell line (CCL-1). **Results**: The rheological properties of these hydrogels show suitable mechanical properties for dermal application, with G′ values of ~10 kPa. From the rheological data, the mesh size of these hydrogels was found to be influenced by the type of the IPN and its composition, varying between 6.5 and 50 nm. The loading capacity of both IPN types and DEX entrapment efficiency were highly influenced by the IPN’s composition. The loading capacity of the IPNs can reach ~3.5%, with a DEX entrapment efficiency of ~35%. The PHEMA/PDMAM IPNs demonstrate an extended release profile with up to ~95% DEX released in 24 h, while PDMAM/PHEMA IPNs release no more than ~25% DEX in 24 h. The drug release profiles follow either non-Fickian diffusion (n~0.6) or case-II transport (n~0.9–1), depending on the IPN’s composition. The PHEMA/PDMAM-based materials were found to be non-cytotoxic against HUT-78 and CCL-1 cells. **Conclusions**: The study reveals that the IPNs of PHEMA and PDMAM appear to be suitable platforms for dermal delivery of dexamethasone as they have appropriate mechanical properties, providing tools to control drug loading and release, and they are biocompatible with human skin cells.

## 1. Introduction

Hydrogels are three-dimensionally (3D) crosslinked polymer networks that are highly swollen in water. Their ability to absorb and retain high amounts of water defines their biocompatibility and hence their important applications in medicine and pharmacy, especially in drug delivery, tissue engineering, sensing, etc. [1]. An advanced and versatile approach for polymer material synthesis is the formation of interpenetrating polymer networks (IPNs) [2]. They consist of two or more polymer networks, mutually interlacing at the molecular scale, without covalent bonding between them (Scheme S1, Supplementary Information). They have many advantages as drug delivery systems, such as easily controllable degree of drug release via tunning the cross-linking density of both networks, creation of phase-separated structure via appropriate hydrophilic/hydrophobic balance of both components, programmable smart behavior (i.e., predictable response upon external stimuli changes, which could enable on–off drug release), etc., and all of these tools are able to modify the drug release profile simply by varying the IPN’s composition [3]. These three key factors define IPNs as suitable platforms for drug delivery—the possibility for control over the IPN’s density and the morphology (their phase-separated structure when formed via the sequential method) determines the tunable drug diffusion rate in and out of their structure, while the possibility for choosing the IPN’s components ensures drug–polymer interactions for precise control over the drug release rate [4].

Dermal drug delivery is focused on the systematic delivery of drug substances via suitable dosage forms applied onto the skin. This approach represents a non-invasive alternative to, e.g., the intravenous route, thus minimizing the discomfort of the patient, the complications related to disruption of the skin’s integrity [5], as well as minimizing the first-pass metabolism and side effects of drugs. In this context, hydrogels exhibit superior properties as dermal drug delivery systems, such as high drug-loading capacity, tunable drug release profiles, water-retaining properties, biocompatibility, controllable swelling [6], etc.

Dexamethasone (DEX) is a synthetic glucocorticoid with anti-inflammatory and immunosuppressive properties. DEX is used in the therapy of several disorders, such as arthritis, allergies, psoriasis, ocular diseases, uveitis, etc. [7]. DEX is available in various dosage forms for different modes of administration, such as oral tablets with doses ranging from 0.5 mg to 40 mg, as injections (under its water-soluble form, dexamethasone sodium phosphate), as topical gels, etc. DEX, however, possesses several disadvantages, such as side effects including elevated blood pressure, changes in mood and behavior, and increased blood sugar levels [8]. Moreover, DEX has a comparatively short half-life, ranging between 2 and 5 h (on averaged ~4 h [9]), high plasma protein binding (nearly 67%), hepatic first pass effect, and can cause gastric irritation upon oral administration. As its systematic effect requires frequent drug uptake, the formulation of an advanced dermal delivery system is a possible approach to overcome all of these disadvantages, which makes DEX a promising candidate for dermal drug delivery. In this respect, two different approaches, such as the development of cubosomes [10] and microneedles [11], have been explored so far for ensuring controlled dermal delivery of DEX. Nevertheless, a limited number of studies highlight the potential of hydrogels as dermal delivery systems for dexamethasone.

Poly(2-hydroxyethyl methacrylate) (PHEMA) is a non-toxic, biocompatible, and hydrophilic polymer specially designed for the development of soft contact lenses by Wichterle and Lim [12]. These lenses are known to provide comfort to the patient as PHEMA is flexible, soft, and ensures an optimal concentration of the drug as well as the necessary residence time to maximize the drug’s therapeutic effects [12]. Besides its ophthalmic application, PHEMA also possess potential for wound dressing development [13] due to its biocompatibility and non-toxicity.

Poly(N,N-dimethylacrylamide) (PDMAM) is a highly hydrophilic, biocompatible, and neutral polymer [14], which in combination with PHEMA (e.g., in an IPN) provides the possibility for precise control over the swelling capabilities and drug delivery performance of the resulting materials [15].

Poly(ethyleneglycol diacrylate) (PEGDA) is a bifunctional ester of ethylene glycol and acrylic acid, which is favorable in terms of its high cytocompatibility, non-toxicity, and the possibility of being easily utilized in the fabrication of drug delivery scaffolds with various shapes and for different applications [16]. This makes it suitable also as curing agent for innovative manufacturing techniques, such as 3D printing [17].

In our previous research, we investigated the usage of poly(2-hydroxyethyl methacrylate) (PHEMA) and poly(N,N-dimethylacrylamide) (PDMAM) interpenetrating polymer networks (IPNs) as drug delivery system of the water-soluble form of dexamethasone—dexamethasone disodium phosphate (DXP). We have found that both drug loading and drug release are diffusion-controlled processes, where the PHEMA/PDMAM ratio is the key factor to controlling them. Moreover, we have shown that DXP is released through Fickian diffusion following a zero-order release kinetics model [15]. Recently, Georgieva et al. created multicomponent IPNs composed of PDMAM-co-poly(ethylene glycol) copolymer and poly(N-isopropylacrylamide), crosslinked with poly(ethylene glycol) diacrylate, and successfully utilized them for dermal delivery of dexamethasone phosphate [18]. However, the potential benefits of IPN-based hydrogels for dermal delivery of DEX remain largely unexplored.

The goal of our study is to explore the potential of PHEMA- and PDMAM-based IPN hydrogels for dermal delivery of water-insoluble DEX. To this purpose, two types of IPNs, namely PHEMA/PDMAM and PDMAM/PHEMA IPNs, were synthesized via the sequential method. The properties of both IPNs, such as their swelling capacity, rheological properties, mesh size, as well as cytotoxicity, were evaluated. Both IPN types were loaded with DEX and their release performance was investigated. The study outlines the relationship between the IPNs’ properties and their behaviors as systems for DEX release.

## 2. Materials and Methods

### 2.1. Materials

2-Hydroxyethyl methacrylate (≥99%) (HEMA), N,N-dimethylacrylamide (99%) (DMAM), poly(ethylene glycol) diacrylate (PEGDA, average Mn 575), 1-hydroxycyclohexyl phenyl ketone (99%) (HCHPK), and dexamethasone (≥98%, HPLC, powder) were obtained from Sigma-Aldrich (St. Louis, MO, USA) (Scheme S2, Supplementary Information). Potassium chloride (KCl), sodium chloride (NaCl), and sodium phosphate dibasic (Na_2_HPO_4_) were purchased from Buchs, Switzerland. All chemicals were used as received without additional purification.

### 2.2. Synthesis of PDMAM/PHEMA IPNs

The PDMAM/PHEMA IPNs (straight IPNs) were prepared using a sequential two-step method. In the first step, single networks (SNs) of PDMAM were synthesized via UV-photopolymerization. To this purpose, an AnalytikJena UV-A lamp, 365 nm, with 1.2 W UV output was used to irradiate the casted solution in a petri dish for 20 min in an open air atmosphere, which resulted into total irradiation dose of ~265 J/m^2^ (see Appendix A). DMAM aqueous solutions (3 M and 5 M) with different amounts of the crosslinking agent PEGDA (0.1, 0.4, 1, and 4 mol.%) were photopolymerized using 0.1 mol.% (with respect to the monomer) 1-hydroxycyclohexyl phenylketone (HCHPK, UV absorption maximum at 333 nm) as the photoinitiator. The obtained SNs were extensively washed to remove residuals and PDMAM SNs with different crosslinking degrees were obtained (Table S1, Supplementary Information).

We optimized the PDMAM SN synthesis with respect to the hydrogel’s mechanical behavior (Table S1, Supplementary Information). Higher DMAM (monomer) and PEGDA (crosslinking agent) concentrations resulted in more brittle and more sticky hydrogels, which made their detachment from the molds difficult. Thus, among the PDMAM SNs listed in Table S1, Supplementary Information, only the SNs obtained using 3 M PDMAM with a low PEGDA concentration (respectively 0.1 and 0.4 mol.%) were strong and elastic enough (Figure S1, Supplementary Information), thus appearing suitable for IPN synthesis. These two SNs samples, labeled as D01 and D04, respectively, were used for the further IPN formation (Figure S1).

The degree of conversion (DC) of DMAM to PDMAM was determined based on the analysis of the DMAM concentration in the wastewater collected during their purification in distilled water. To this purpose, the calibration curve for the monomer DMAM obtained in a previous study [15] was applied. The degree of conversion of the monomer DMAM to polymer (PDMAM) in the case of D01 and D04 SNs was estimated to be ~90 ± 7%, based on measurements of six different PDMAM SNs from each type of SN.

In the second step, the dry PDMAM SNs were swollen for 72 h at room temperature in aqueous HEMA solutions containing also PEGDA (0.1 mol.%) and HCHPK (0.1 mol.%). The swollen SNs were exposed to UV light (365 nm) for 20 min to facilitate the in situ crosslinking polymerization of HEMA to PHEMA within the PDMAM SNs. The resulting IPNs were thoroughly washed with distilled water, which was replaced daily, to ensure the complete removal of residuals.

The DC of HEMA to PHEMA during the 2nd stage of the IPN synthesis was estimated to be ~93 ± 4%, using again the HEMA concentration in the wastewater obtained during the IPN purification step. To this purpose, the calibration curve for the monomer HEMA from our previous study [15] was used.

The composition of the PDMAM/PHEMA IPNs was expressed as the weight part of PDMAM in the IPNs ($r_{D}$), determined using Equation (1):

(1) $$r_{D}=\frac{m^{\mathrm{PDMAM}}}{m^{\mathrm{PHEMA}}+m^{\mathrm{PDMAM}}}$$

where m^PDMAM^ and m^PHEMA^ are the weights of the PDMAM and PHEMA SNs in the IPN, respectively [15].

The PDMAM/PHEMA IPN sample descriptions are presented in Table 1, while the appearance of the samples (in dry and swollen state) is presented in Figure S2, Supplementary Information.

### 2.3. Synthesis of the Reverse PHEMA/PDMAM IPNs

The synthesis of the reverse IPNs, namely PHEMA/PDMAM IPNs, which differ from the straight type by the order of SN formation, is described elsewhere [15]. Briefly, SNs of PHEMA were synthesized via bulk UV-initiated crosslinking photopolymerization of the PHEMA monomer, using PEGDA as the crosslinking agent (1 mol.%) and the photoinitiator HCHPK (0.1 mol.%). The polymerization took place for 20 min under UV light at 365 nm at room temperature. After removing residuals and drying, the single networks (SNs) were swollen in aqueous solutions of PDMAM monomer containing PEGDA (0.1 mol.%) and HCHPK (0.1 mol.%) (Table 2). After 72 h, the swollen SNs were irradiated with UV light (365 nm, 20 min) to facilitate the in situ crosslinking polymerization of DMAM within the PHEMA SN. The resulting IPNs were washed with distilled water until no residuals were detected in the UV spectrum (JASCO V-730 UV/Vis spectrophotometer, Jasco, Tokyo, Japan). Their appearance is shown in Figure S3, Supplementary Information.

The degrees of DMAM and HEMA conversion to the respective polymers during PHEMA/PDMAM IPN synthesis were found to be, respectively, ~92 ± 5% for DMAM and ~96 ± 2% and HEMA, as described elsewhere [15].

### 2.4. Rheological Properties

The rheological study was performed using a ThermoHaake RheoStress 600 rheometer (Thermo Fisher Scientific, Waltham, MA, USA) with parallel plate geometry (20 mm diameter and 1 mm gap) at 25.0 ± 0.1 °C. A stress amplitude of 1 Pa was selected for the frequency scan tests conducted in the range of 0.1–100 Hz.

The elastic modulus obtained from the rheological measurements was used to determine the mesh size of the hydrogel network using Equation (2) [19]. In this equation, G′ represents the storage modulus in the plateau region from the frequency sweep experiment, R is the ideal gas constant (J.mol^−1^K^−1^), T is temperature in Kelvin, and N_A_ is Avogadro’s constant. L is the side length of the cubic-shaped volume element of the polymer network.

(2) $$L=\xi ={\left(\frac{\mathrm{RT}}{G^{\prime }N_{A}}\right)}^{\frac{1}{3}}$$

The results presented are obtained after averaging the data obtained from the measurements performed for three different samples (n = 3).

### 2.5. Swelling Properties

#### 2.5.1. Equilibrium Swelling Ratio (ESR)

The equilibrium swelling ratio (ESR) of SNs and IPNs was measured by immersing dry disk-shaped samples in water until they reached a constant weight. The ESR was calculated using Equation (3), where $m^{\mathrm{sw}}$ and $m^{\mathrm{dry}}$ represent the weights of the swollen and dry samples, respectively. The results presented were obtained after averaging the data obtained for three different pieces from one sample (n = 3).

(3) $$\mathrm{ESR}=\frac{m^{\mathrm{sw}}-m^{\mathrm{dry}}}{m^{\mathrm{dry}}}$$

#### 2.5.2. Determination of ESR in Ethanol

The ESR of SNs and IPNs was determined in 99.8% ethanol (EtOH) because ethanol is a solvent for DEX loading. The ESR in EtOH was calculated by using Equation (3), where m^sw^ and m^dry^ are the weight of the sample in its swollen (in ethanol) and dry state, respectively. The results presented were obtained after averaging the data obtained for three different pieces from each sample (n = 3).

#### 2.5.3. Ionic Strength (IS) Responsiveness

To evaluate the responsiveness of the SNs and the IPNs to the ionic strength (IS) of the media, dry disk-shaped samples were immersed in NaCl aqueous solutions with varying concentrations (respectively 0.001 M, 0.01 M, 0.1 M, 1 M, 2 M, and 5 M) for 24 h. The equilibrium swelling ratio (ESR) was calculated using Equation (3), where $m^{\mathrm{sw}}$ and $m^{\mathrm{dry}}$ are the weights of the swollen and dry samples, respectively. The results presented were obtained after averaging the data obtained for three different pieces from each sample (n = 3).

#### 2.5.4. Temperature Responsiveness

The effect of temperature on the ESR of the PDMAM SNs and PDMAM/PHEMA IPNs was evaluated by swelling dry disk-shaped samples for 6 h in water at different temperatures, ranging from 20 to 55 °C. The ESR was calculated using Equation (3), where m^sw^ and m^dry^ are the weight of the sample swollen in water for 6 h at the respective temperature and in a dry state, respectively. The results presented were obtained after averaging the data obtained for three different pieces from each sample (n = 3).

### 2.6. Scanning Electron Microscopy (SEM)

The morphology of the fractured surfaces of dry SN and IPN samples was analyzed using a Lyra 3 XMU scanning electron microscope (Tescan, Brno, Czech Republic) operating at 10 kV. Prior to examination, the fractured surfaces of the dry samples were coated with a thin carbon film (~10 nm).

### 2.7. Cell Lines and Culture Conditions

Normal murine fibroblast cells (CCL-1) and human cutaneous T-cell lymphoma cells (HUT-78) were obtained from the German Collection of Microorganisms and Cell Cultures (DSMZ GmbH, Braunschweig, Germany). The cell cultures were maintained in the specified media: EMEM with 10% horse serum for CCL-1 cells and IMDM with 20% fetal bovine serum for HUT-78 cells. Cells were incubated at 37 °C in a 5% CO_2_ humidified atmosphere as per standard cell culture conditions.

### 2.8. In Vitro MTT Colorimetric Assay

In prior experiments, the samples were ground in a mortar with the aid of liquid nitrogen to a fine powder. The powders were UV-sterilized using a UV-C germicidal lamp (254 nm for 30 min) (MRL-58 Multi-Ray lamp, AnalytikJena, Upland, CA, USA).

The biocompatibility and in vitro cytotoxicity of the polymeric materials were evaluated using a validated methodology for assessing cell viability, known as the Mosmann MTT method. Exponential phase cells were harvested and seeded (100 μL/well) in 96-well plates at the appropriate density (3 × 10^5^ for the suspension culture HUT-78 and 1.5 × 10^5^ for the adherent CCL-1 cells). Following a 24 h incubation, cells were exposed to two different doses of the polymer samples, equivalent to 20 mg/mL and 16 mg/mL final concentrations in the well. After an exposure time of 72 h, filtered and sterilized MTT substrate solution (5 mg/mL in PBS) was added to each well of the culture plate. A further 1–4 h incubation allowed for the formation of purple insoluble formazan crystals. The latter were dissolved in isopropyl alcohol solution containing 5% formic acid prior to absorbance measurements at 550 nm using a microplate reader (Labexim LMR-1), Labexim International, Prague, Czech Republic). The collected absorbance values were blanked against MTT-isopropanol solution and normalized to the mean value of the untreated control (100% cell viability).

### 2.9. Drug Loading

The SNs and IPNs were loaded with DEX by swelling dry samples in an ethanol solution of DEX with concentrations of 2.5, 7.5, and 15 mg/mL. After being dried and stored in sealed zipper bags, disk-shaped samples with diameter of ~4.5 mm were weighed on analytical scales and then placed in the respective DEX-ethanol solution for 72 h. Due to the susceptibility of dexamethasone to light, the solutions and the drug-loaded samples were stored in a light-protected environment. The loaded samples were dried from the ethanol at ambient conditions, sealed in zipper bags, and stored in a light-protected environment.

The entrapment efficiency (EE) and drug loading (DL) were indirectly determined by measuring the UV absorbance of the remaining drug solution after the loading process. Aliquots of 50 µL were diluted with ethanol and their UV absorption was measured at 242 nm. The EE and DL were calculated from the linear regression of the calibration curve, preliminary obtained for the DEX solution in ethanol (Equation (4)). To this purpose, three series of DEX solutions in ethanol, with concentrations in the range of 2.5 µg/mL to 17 µg/mL, were prepared and the UV absorbance of each solution was measured at 242 nm. The linear regression of the DEX calibration curve in ethanol (Equation (4)) was found to be:

ABS_242_ = 35.67 × C_DEX_ + 0.18 (R^2^ = 0.99922) (4)

where C_DEX_ and ABS_242_ are, respectively, the DEX concentration and its UV absorbance, measured at 242 nm.

EE and DL were calculated using Equations (5) and (6), respectively:

(5) $$\mathrm{EE}=\frac{m_{\mathrm{sample}}^{\mathrm{drug}}}{m_{\mathrm{total}}^{\mathrm{drug}}}\times 100\%$$

(6) $$\mathrm{DL}=\frac{m_{\mathrm{sample}}^{\mathrm{drug}}}{m_{\mathrm{nonloaded}}^{\mathrm{sample}}}\times 100\%$$

where $m_{\mathrm{sample}}^{\mathrm{drug}}$ is the drug amount loaded in one polymer sample, $m_{\mathrm{total}}^{\mathrm{drug}}$ is the total drug amount in the initial solution used for loading, and $m_{\mathrm{nonloaded}}^{\mathrm{sample}}$ is the weight of the dry polymer sample before drug loading. The results were averaged from the data obtained for three pieces from the respective sample (n = 3).

### 2.10. In Vitro Drug Release

The drug release tests were carried out in 12 mL Franz diffusion cells using disk-shaped samples with a diameter of ~10 mm, loaded with ~ 1 mg DEX. After complete evaporation of ethanol from the samples, loaded in DEX ethanol solution, they were rehydrated (swollen) in water until reaching their equilibrium swelling rate and placed in the donor compartment of the Franz cells. The donor compartments were separated with nylon membranes (0.22 μm pore-size) from the acceptor compartments. The cells were sealed and filled to the mark with 20% EtOH in PBS as the acceptor phase and then placed in the STEM device (HDT 1000 Vertical Diffusion Cell Test System, Copley, Nottingham, UK) at 32 °C, 800 rpm. At defined time intervals, the entire acceptor phase was changed with fresh portions to maintain sink conditions, and their UV absorbance was measured at 242 nm. The amount of the released drug was calculated using the calibration curve for the respective drug in 20% EtOH in PBS media. These calibration curves were different from the ones obtained for the purpose of drug loading evaluation, as here PBS with added ethanol was used instead of the pure ethanol used during the loading process.

For obtaining the calibration curve of DEX in PBS with added ethanol, 1 mg/mL stock solution in ethanol was used to obtain a series of solutions in 20% EtOH in PBS with concentrations in the range of 0.5–15 μg/mL. The UV absorbance of these solutions was measured at 242 nm and the linear regression equation of the calibration curve was found to be:

ABS_242_ = 0.029 × C_DEX_ + 0.061 (R^2^ = 0.99367) (7)

The results were obtained for one piece from the respective SNs or IPNs, i.e., for each IPN’s composition.

### 2.11. DEX Release Kinetics

The DEX release profiles were analyzed using the following mathematical models:

- Zero-order (ZO)—this model assumes that the drug is released from the carrier at a constant rate, i.e., this is a concentration-independent model [20]:
  (8) $$Q_{t}=Q_{0}+k_{0}\times t$$
- First order (FO)—this model presumes that the rate of change in the drug concentration with time is directly proportional to the amount of the drug still remaining in the delivery system, i.e., this is a concentration-dependent model [21]:
  (9) $${\mathrm{logQ}}_{t}={\mathrm{logQ}}_{i}-k_{1}\times \frac{t}{2.303}$$
- Higuchi model (HM)—this model describes drug release as a diffusion-driven process governed by Fick’s law, with a drug release rate dependent on the square root of time [21]:
  (10) $$Q_{t}=k_{H}\times {\;t}^{0.5}$$
- Korsmeyer—Peppas model (KP)—this model is applied when the release mechanism is unclear or when multiple drug release phenomena are involved [20]. Very often, this model appears to be appropriate for drug diffusion in hydrogels based drug delivery systems:
  (11) $$\frac{Q_{t}}{Q_{\infty }}=k_{\mathrm{KP}}\times t^{n}$$

Here, $Q_{t}$ and $Q_{0}$ represent the amounts of the drug released at time t and at the start of the release experiment, respectively; $Q_{i}$ and $Q_{\infty }$ are the amounts of the drug loaded into the sample. $k_{0}$, $k_{1}$, $k_{H}$, and $k_{\mathrm{KP}}$ are kinetic constants, while n is the diffusional exponent, which is indicative of the release mechanism: when n ≤ 0.45, Fickian diffusion of the drug takes place; when 0.45 ≤ n ≤ 0.89, drug transport follows Non-Fickian diffusion; and when n > 0.89, the drug follows complex transport (case-II) [22].

## 3. Results

### 3.1. Rheological Properties

**Figure 1:**
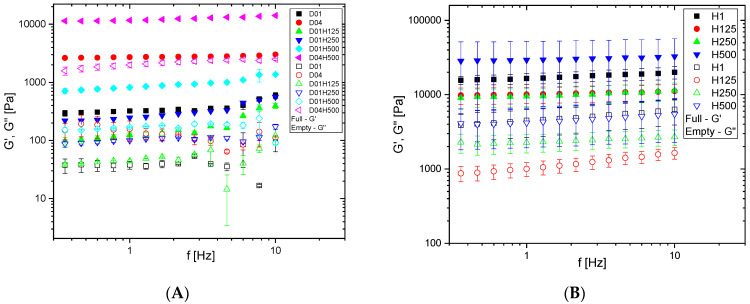
Storage modulus (G′) (full symbols) and loss modulus (G″) (empty symbols) for the straight PDMAM/PHEMA IPNs (**A**) and for the reverse PHEMA/PDMAM IPNs (**B**) obtained via frequency sweep experiments.

The frequency dependences of the shear storage (G′) and loss (G″) moduli of both types of IPNs, the straight PDMAM/PHEMA and the reverse PHEMA/PDMAM IPNs, are presented in Figure 1A and Figure 1B, respectively.

#### 3.1.1. Rheology of the Straight PDMAM/PHEMA IPNs

The higher crosslinking agent concentration for the starting SNs, namely PDMAM, resulted in one order of magnitude higher storage modulus: the G′ values increased from ~0.5 kPa for 0.1 mol.% PEGDA (D01) to ~1.5 kPa when the PEGDA concentration increased to 0.4 mol.% (D04). Thus, the network density expectedly influenced the mechanical properties of the starting PDMAM single networks (Figure 1A).

The inclusion of PHEMA within the PDMAM SNs after IPN formation resulted in a G′ value increase from ~1.5 kPa to ~10 kPa for D04H500. This was related to the increased number of entanglements in the IPN’s structure, which acted as physical junctions and increased the mechanical performance (Scheme S1, Supplementary Information).

In general, for all examined SN and IPN samples in Figure 1A, G′ was more than one order of magnitude higher than G″, which meant that these hydrogels were stable and had sufficient mechanical strength to be stand-alone materials.

#### 3.1.2. Rheology of the Reverse PHEMA/PDMAM IPNs

The mechanical properties of the reverse PHEMA/PDMAM IPNs were not influenced by their composition in a clear way (Figure 1B). Their storage modulus (G′) was ~10 kPa, which corresponded very well with previously reported values [23] and it was nearly one order of magnitude higher than their loss modulus (G″), which was ~1.5 kPa. The higher G′ value of the IPN PHEMA/PDMAM H500 ($r_{D}^{r}$ = 0.31) could be attributed to the higher crosslinking density of this IPN, defined by the incorporation of higher amounts of the second component, PDMAM, which resulted in increased numbers of entanglements as well as interactions between both component networks. This effect was similar to the effect of the increased crosslinking density observed for poly(N,N-diethyl acrylamide-co-N,N-dimethylacrylamide) hydrogels crosslinked with polyethylene glycol 600 dimethacrylate [24], where similar reasons for their improved mechanical performance were assumed.

### 3.2. IPNs’ Mesh Size

**Figure 2:**
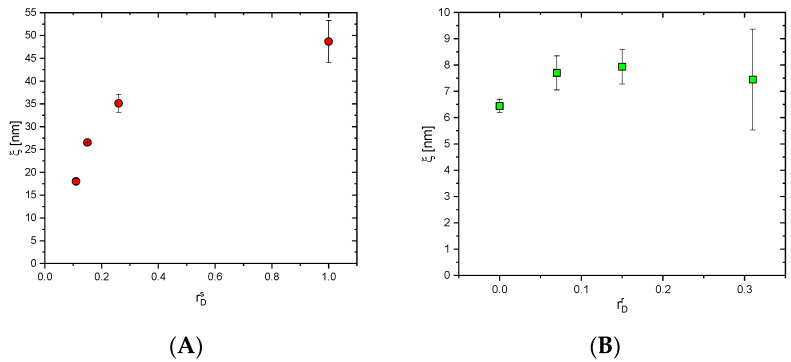
Mesh sizes (ξ) of (**A**) PDMAM/PHEMA IPN and (**B**) PHEMA/PDMAM IPN hydrogels as a function of PDMAM content.

The mesh sizes of PHEMA/PDMAM and PDMAM/PHEMA IPNs in a swollen state were calculated using Equation (2) (Figure 2).

#### 3.2.1. Mesh Size of the Straight PDMAM/PHEMA IPNs

The mesh size of the PDMAM/PHEMA IPNs decreased as the content of the 2nd component, PHEMA, increased, i.e., the PDMAM content decreased. While the PDMAM SNs (PDM, Table 1, $r_{D}^{s}$ = 1.0) had a mesh size of ~50 nm, the IPN with the highest PHEMA content ($r_{D}^{s}$ = 0.11) had a mesh size of ~18 nm (Figure 2A). This trend could be attributed to the higher hydrophobicity of PHEMA as compared to PDMAM, which reduced the swelling ratio of the PDMAM/PHEMA IPNs when the PHEMA content increased. Moreover, the physically entangled structure of the IPNs as compared to the SNs additionally reduced the swelling ability, as also detected by the rheological studies of the same networks.

#### 3.2.2. Mesh Size of the Reverse PHEMA/PDMAM IPNs

The mesh size of the PHEMA SN was evaluated to be ~6.5 nm, which correlated with the reported value by Bengani et al. of 2.98 nm for PHEMA crosslinked with ethylene glycol dimethacrylate (EGDMA) with a similar crosslinking agent amount [25]. The slightly higher mesh size obtained in this work could be attributed to the longer chain length (i.e., molecular weight) of the crosslinking agent used here, namely PEGDA, as compared to EGDMA used in the reference study. The incorporation of PDMAM within the PHEMA networks resulted in an increase in the mesh size of the IPNs to ~8 nm, most probably due to the more hydrophilic nature of the 2nd network of PDMAM (Figure 2B). However, the effect of the 1st network’s nature was significant and the reverse IPNs had much lower mesh sizes as compared to the straight ones due to the hydrophobic nature of PHEMA as compared to PDMAM.

### 3.3. Swelling Properties

#### Equilibrium Swelling Ratio (ESR)

**Figure 3:**
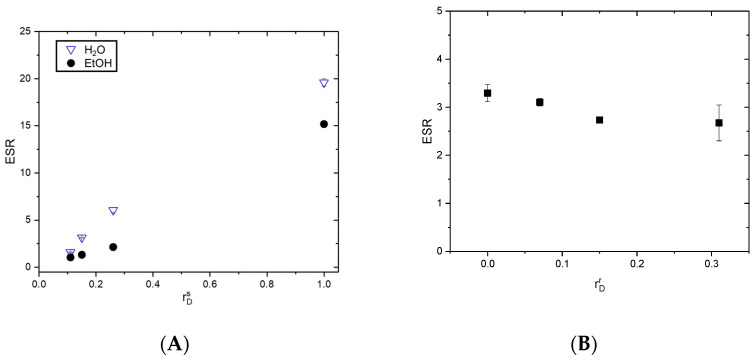
ESR for PDMAM/PHEMA IPNs synthesized using PDMAM SNs with 0.1 mol.% PEGDA in water and in ethanol (EtOH) (**A**) and ESR for PHEMA/PDMAM IPNs in EtOH (**B**).

The ESR values of the PDMAM/PHEMA IPNs are shown in Figure 3A. As can be seen, the ESR for the IPN synthesized from PDMAM with 0.1 mol.% PEGDA (D01) as the first network was significantly influenced by its composition. For the PDMAM SNs, the ESR value was ~20, and as the PHEMA amount rose ($r_{D}^{s}\;$ decreased), the ESR value dropped to ~2.3. This was attributed to two factors: (i) the increase in the amount of the component with higher hydrophobicity and thus with lower swelling capacity, PHEMA (ESR of ~0.5 for PHEMA SN as was determined in our previous work [15]), and (ii) the increased network density of IPNs as compared to the respective SNs. As the results showed, the ESR in EtOH followed the same trend as in water, but with decreased values for ESR. This confirmed that the IPN’s composition was a key factor in controlling its swelling ability.

**Figure 4:**
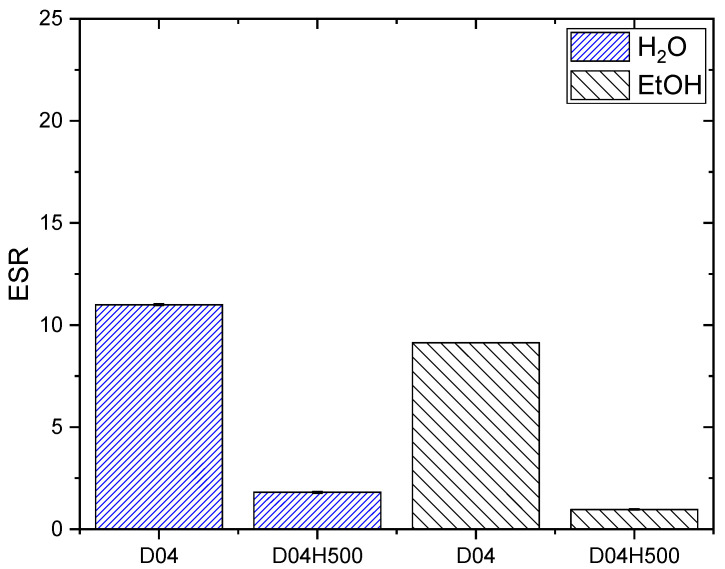
ESR of PDMAM SN with 0.4 mol.% PEGDA (D04) and its IPN with PHEMA (D04H500), in water and in ethanol (EtOH).

The PDMAM SN with 0.4 mol.% PEGDA (D04) demonstrated a lower ESR of ~11 as compared to D01, which could be attributed to the increased network density as the crosslinking agent concentration increased (Figure 4). The IPN synthesized from D04 SN (e.g., D04H500 in Figure 4) demonstrated a lower ESR of ~2.4 as compared to the IPN of D01 due to the effects of both PHEMA inclusion and the increased network density of the PDMAM SNs. Similarly, in EtOH, the ESR for IPN D04H500 was lower than the ESR for D04, which was explained again by the increased network density due to the IPN structure (Scheme S1, Supplementary Information).

On the other hand, the ESR values of the PHEMA/PDMAM IPNs in EtOH were not strongly influenced by their composition and were all ~3 (Figure 3B), which was higher than the ESR values in EtOH determined for the PDMAM/PHEMA IPNs. Here, the effect of the 1st network’s nature (PHEMA was the component with higher hydrophobicity and lower swelling capacity) was highly pronounced and strongly defined the IPN’s behavior.

### 3.4. Scanning Electron Microscopy (SEM)

**Figure 5:**
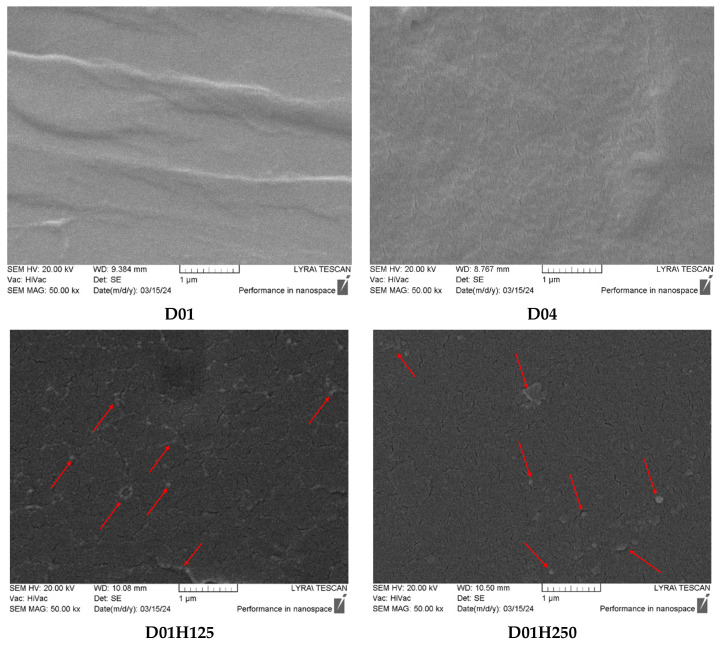
SEM images of fractured surfaces of PDMAM SNs and PDMAM/PHEMA IPNs. Red arrows point at the second phase domains of PHEMA.

SEM was used to investigate the morphology of fractured surfaces of the PDMAM SNs and PDMAM/PHEMA IPNs (Figure 5). Both PDMAM SNs, synthesized with 0.1 mol.% PEGDA (D01) and with 0.4 mol.% PEGDA (D04), revealed smooth homogenous morphologies. For the PDMAM/PHEMA IPNs, the inclusion of PHEMA gave rise to domains of the second network (i.e., phase separation occurred), which was typically observed for IPNs synthesized through the sequential method. Our SEM study on the PHEMA/PDMAM IPNs showed that these IPNs were not phase-separated at similar compositions (i.e., similar ratio between both SNs), which was attributed to their better miscibility. So, it could be speculated that, besides miscibility, another important factor that contributes to phase separation was the order of SN formation in the IPNs. As observed by SEM, the microstructural changes in the IPNs were in good agreement with the visual appearance of the samples at the macro level (Figure S2, Supplementary Information). While the PHEMA/PDMAM IPNs, which were homogenous, appeared to be transparent, the PDMAM/PHEMA IPNs, which tended to be phase-separated, appeared to be turbid (Figure S2).

### 3.5. Entrapment Efficiency (EE) and Drug Loading (DL) of Dexamethasone

**Figure 6:**
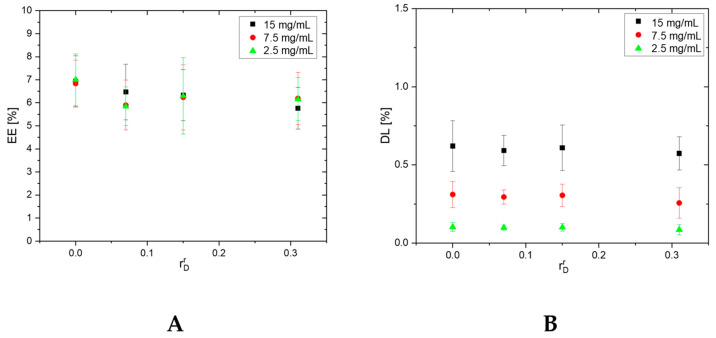
Dependence of DEX EE (**A**) and DL (**B**) in PHEMA SN and PHEMA/PDMAM IPNs as a function of their composition ($r_{D}^{r}$).

The EE and DL of DEX in the PHEMA/PDMAM IPNs are presented in Figure 6. As can be seen, the EE was not affected by either the concentration of the DEX solution used for the drug loading experiments or by the IPN’s composition, and it was ~7% for all IPN compositions. This result corresponded very well with the results obtained for the ESR in ethanol (Figure 3A), as DEX loading was carried out by diffusion from its ethanol solution. The results for DL of the PHEMA/PDMAM IPNs with DEX showed that the DL depended only on the DEX concentration in the loading solution, and it was not affected by the IPN’s composition, which was again well related to the IPN’s swelling behavior in ethanol.

**Figure 7:**
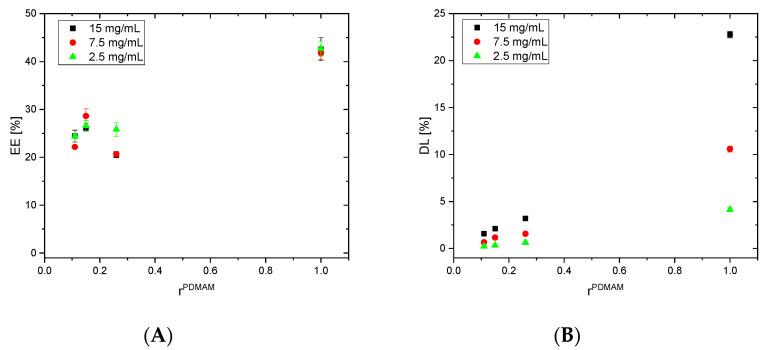
Dependence of DEX EE (**A**) and DL (**B**) in PDMAM SNs and PDMAM/PHEMA IPNs, synthesized using PDMAM SNs with 0.1 mol.% PEGDA as a function of their composition ($r_{D}^{s}$).

Similarly, the EE and DL of DEX in the PDMAM/PHEMA IPNs and PDMAM SNs depended entirely on the swelling capacity and the DEX concentration in the loading solution, as DEX loading was driven by drug passive diffusion (Figure 7).

### 3.6. In Vitro Drug Release of DEX

**Figure 8:**
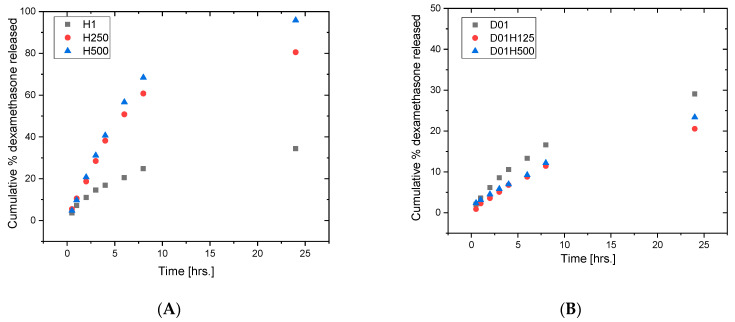
Drug release profiles of DEX from PHEMA SN (H1) and PHEMA/PDMAM IPNs (H250 and H500) (**A**) and from PDMAM SN (D01) and PDMAM/PHEMA IPNs (D01H125 and D01H500) (**B**).

The in vitro release profiles of DEX from the PHEMA SN and PHEMA/PDMAM IPNs are presented in Figure 8A. As can be seen, no burst release effect was observed for DEX in any of the obtained release profiles. The PHEMA/PDMAM IPNs demonstrated an extended release profile with up to ~95% DEX released as compared to ~30% for the PHEMA SN. This was most likely due to the increased swelling capacity of the IPNs as compared to the PHEMA SN, which was defined by the favored permeability of dexamethasone due to the more hydrophilic nature of the PDMAM network. Thus, the PHEMA/PDMAM IPNs demonstrated good potential for prolonged release of hydrophobic drug substances in general, and in particular of dexamethasone.

The PDMAM/PHEMA IPNs demonstrated retarded DEX release profiles, where only ~10–15% DEX was released during the first 8 h, and no more than ~30% DEX was released in the first 24 h (Figure 8B). The effect of the increased network density in the straight IPNs was clearly visible as the PDMAM SNs released DEX more rapidly as compared to the respective IPNs.

For both IPNs, the DEX release profiles differed as compared to the DEX release profiles obtained for the respective single networks, which demonstrated the contribution of the specific IPN’s structure and related performance as a drug delivery system.

### 3.7. Dexamethasone Release Kinetics

The obtained data showed that the release of DEX from the PHEMA SN and PHEMA/PDMAM IPNs was best described by the Higuchi model, i.e., the model valid for drug diffusion through a hydrogel matrix (Table 3). The obtained data also showed a good correlation with the zero-order kinetics model, i.e., a time-dependent drug release profile. Thus, the composition and order of formation of the SNs in the IPNs provide the tools to develop systems for precise control over the rate and extent of dexamethasone release. Here, it must be noted the relatively high values for the diffusional exponent n obtained for the release profiles from the PHEMA/PDMAM IPNs, namely n ~1.0, which pointed out that the drug transport was case-II transport (n > 0.89), i.e., it was rather controlled by polymer chain relaxation.

DEX release from the PDMAM SNs was best described by the Higuchi model, showing again typical drug diffusion through a hydrogel matrix. This was also confirmed by the diffusional exponent obtained from the Korsmeyer–Peppas model (n = 0.582), which indicated a drug diffusion mechanism close to Fickian diffusion. As the results in Table 3 show, DEX release from the PDMAM/PHEMA IPNs was achieved through non-Fickian diffusion (0.45 ≤ n ≤ 0.89), where also polymer chain relaxation started to influence drug molecule transport through the polymer matrix.

### 3.8. In Vitro MTT Colorimetric Assay

**Figure 9:**
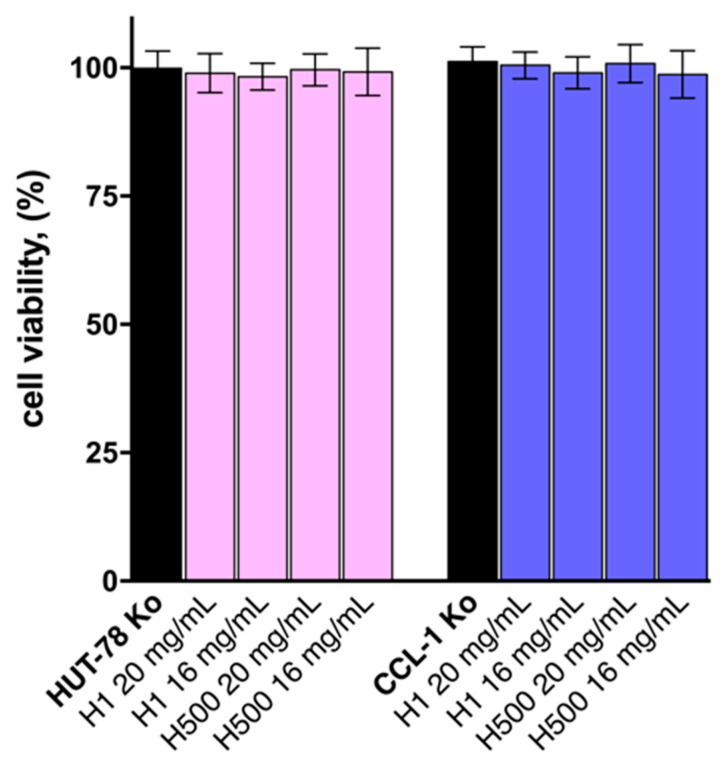
Cell viability of cutaneous T-cell lymphoma cells (HUT-78) and normal murine fibroblast cell line (CCL-1) following 72 h exposure to different concentrations of H1 and H500 PHEMA/PDMAM IPNs. All experiments were run in triplicate and data are expressed as the mean ± SD. Statistical significance of the data was assessed using a one-way ANOVA (*p*-value ≤ 0.05 was considered statistically significant).

The in vitro effect and biocompatibility of the unloaded H1 and H500 polymer carriers on cutaneous T-cell lymphoma cells (HUT-78) and the normal murine fibroblast cell line (CCL-1) are demonstrated in Figure 9. As evident from the figure, neither polymer system exerted any statistically significant effect on cell viability in both in vitro-tested models, despite their exposure to concentrations as high as 20 mg/mL in plate wells. Cell viability and their propensity for growth after 72 h exposure were comparable to the control group, and no morphological changes in cell appearance with regard to their size, shape, membrane integrity, detachment, and cell-to-cell contacts were observed. According to the obtained results, the screened polymeric carriers emerged as a feasible and suitable technological approach for the intended transdermal drug delivery.

## 4. Discussion

This study explores the potential of straight PDMAM/PHEMA and reverse PHEMA/PDMAM IPN hydrogels for dermal delivery of the hydrophobic drug dexamethasone. The order of formation of the IPN’s structure determines the swelling capacity of the obtained hydrogels. When the PHEMA network is in situ-formed within the PDMAM SNs, this leads to a decrease in the overall capacity of the IPNs to swell due to saturation with a component with lower swelling capacity and higher hydrophobicity (Figure 3A,B). On the contrary, when the PDMAM network is in situ-formed within the PHEMA SN, the overall swelling capacity of the resulting PHEMA/PDMAM IPNs increases due to its higher hydrophilicity [15]. Thus, even when both types of IPNs have relatively similar compositions, i.e., similar ratio between PHEMA and PDMAM networks (Table 1 and Table 2), the swelling capacity of the straight PDMAM/PHEMA IPN hydrogels is higher as compared to that of the reverse PHEMA/ PDMAM IPN hydrogels. This was also found to influence the mesh size of the IPN hydrogels (Figure 2). The calculated size of the DEX molecule is estimated to be ~1.27 nm, which is smaller than the calculated mesh size of both types of IPN hydrogels. This suggests that the drug molecules can freely diffuse within the SN and IPN swollen networks, so their release could be expected to be dominated mainly by their diffusion rate rather than by polymer chain relaxation [26].

It was demonstrated that, for all examined samples, the storage modulus G′ is over one order of magnitude higher than the loss modulus G″ (Figure 1A,B), i.e., all hydrogels are sufficiently mechanically strong and behave as stand-alone materials. The storage moduli of both the straight PDMAM/PHEMA and the reverse PHEMA/PDMAM IPN hydrogels was ~10 kPa, which fits well with the interval from 1 to 100 kPa that is suitable and compatible for human skin related applications [27]. Moreover, the reverse PHEMA/PDMAM IPN hydrogels appear not to be influenced by the ionic strength (electrolyte concentration) of the media [15] up to 0.1 mol/L. By contrast, the ability of the straight PDMAM/PHEMA IPNs to sustain such ionic strength media changes extends to ~2.5 mol/L (Figure S4). Since our hydrogels are intended to be applied to deliver the drug with a predicted rate on the skin’s surface, their sustainable behavior is mandatory. The concentrations of Na^+^ and Cl^−^ in sweat are around 42.9 ± 18.7 mmol·L^−1^ and 32.2 ± 15.6 mmol·L^−1^, respectively [28], but they can be influenced by thermal stress, sweat production rate, as well as by body temperature [29]. The presence of pendant –CH_3_ and –CH_2_CH_2_OH groups originating from PHEMA and pendant –N(CH_3_)_2_ groups from PDMAM determine the temperature responsiveness of the PHEMA/PDMAM [15] and PDMAM/PHEMA IPNs (Figure S5). This is expressed as a nearly linear decrease in their ESR as the temperature increases, instead of a sharp transition, which can be attributed to the random distribution of the pendant groups responsible for this temperature dependence [30]. However, changes in the skin’s surface temperature cannot be considered as a factor that can contribute to altering drug release from these hydrogels as they are not significant enough to provoke such temperature responsiveness.

DEX is insoluble in water and, due to this, both IPN types were loaded with DEX from DEX-ethanol solution. The straight PDMAM/PHEMA IPNs demonstrated better capacity for DEX loading, which was expressed as an EE of ~25%, i.e., 3.5 times higher than the EE for the reverse PHEMA/PDMAM IPNs (Figure 6A and Figure 7A). This effect can be attributed to the increased mesh size of these hydrogels allowing faster diffusion of solutes with smaller sizes [31]. As the results indicate, the process of DEX loading is driven by passive diffusion (Figure 6 and Figure 7) and drug loading is limited by the swelling capacity of the respective IPNs in the loading media, i.e., in this case ethanol. However, higher drug loading could be achieved by, e.g., the introduction of more hydrophobic moieties in the network structure [32], including complex structures such as cyclodextrins [33].

As was demonstrated, DEX release from both types of IPNs is well described by zero-order (time-dependent) release kinetics. For the PHEMA SN, the release rate of DEX was found to be ~27 μg/h, while the presence of the PDMAM component (more hydrophilic and with higher swelling capacity) facilitated drug release and the drug release rate reached ~86 μg/h. For the PDMAM SNs (more hydrophobic and with lower swelling capacity), the rate of DEX release decreased to ~38 μg/h. These results clearly indicate the significance of both factors—the composition as well as the order of SN formation in the IPNs.

DEX release from the reverse PHEMA/PDMAM IPNs follows a complex transport mechanism (case-II transport). Previous work demonstrated that this effect may be observed due to a large increase in osmotic pressure as a driving force when the solvent fronts meet [34]. Another approach proposes that diffusion is hindered by the presence of the polymer mesh [35]. While the second case is valid for higher-molecular-weight solutes, in our study, most probably the reason for the observed type of behavior was the meeting of water as the solvent in the hydrogel matrix and the release media, consisting of 20% EtOH in PBS, which increased the osmotic pressure at the border between the carrier and the release media. Additionally, the solubility of DEX, which is practically insoluble in water, is increased in 20% EtOH-containing media.

DEX release from the straight PDMAM/PHEMA IPNs hydrogels is best described by the Korsmeyer–Peppas model and demonstrates non-Fickian drug diffusion (i.e., anomalous transport), where polymer chain relaxation also influences the drug transport mode. A similar effect is reported, for example, for indomethacin release from PHEMA/MAA hydrogel membranes by Varshosaz and Hajian [36].

An important feature of hydrogels is their high biocompatibility. PHEMA, for example, is reported not to show significant intrinsic surface toxicity against 3T3 cells [37]. However, the cytotoxicity of PHEMA against L929 cells is controversial. While Prasitsilp et al. reported a decrease to 52–65% viability of these cells as compared to the control [38], Zhang et al. reported no observable cytotoxicity of PHEMA toward L929 fibroblast cells [39]. The reported cytotoxic effect could be attributed to the release of nonreacted monomer (HEMA) that eventually damaged fibroblast cells [40]. In addition, the photoinitiator 1-HCHPK (known also as Irgacure 184) was found to be more toxic as compared to other photoinitiators, like Irgacure 2959 [41]. A recent study by Blinova et al. showed that, at concentrations lower than 1 mg/L of 1-HCHPK (known also as Irgacure 184), this compound did not pose a hazard to freshwater microcrustaceans [42]. In summary, this study demonstrates that both the PHEMA SN and PHEMA/PDMAM IPNs are not cytotoxic against HUT-78 and CCL-1 cells, so these materials can be considered as safe for biomedical usage.

Future perspectives of the current study include the design of more complex drug delivery devices, such as microneedle array patches, from PHEMA/PDMAM IPNs for potential transdermal delivery of bioactive substances to the dermal skin layer as well as tests on animals to evaluate their in vivo biocompatibility.

## 5. Conclusions

In this study, the potential of two types of IPN hydrogels based on PDMAM/PHEMA IPNs for dermal drug delivery was demonstrated. The order of IPN synthesis appears to strongly influence the respective IPN properties. For example, the newly synthesized straight PDMAM/PHEMA IPNs had nearly 2.5-fold higher swelling capacity as compared to the reverse PHEMA/PDMAM IPNs. The order of SN formation also affects the rheological properties of the IPNs: while the storage modulus of the straight PDMAM/PHEMA IPNs was ~1 kPa, the reverse PHEMA/ PDMAM IPNs had an almost 10 times higher storage modulus (~10 kPa), which could be partially related to the lower ESR of the latter. The mesh size of the IPN also depends on the IPN composition, and it varied between 6.5 and 50 nm. The different swelling behaviors of both IPNs expectedly influenced their drug-loading capacity as well as their DEX release profiles. The DEX loading from drug ethanolic solution shows a diffusion-controlled mechanism, with the DL and EE values strongly depending on the swelling capabilities of the respective IPNs in ethanol. The DEX release profiles suggest case-II transport of the drug, with n > 0.89, suggesting a complex transport mechanism with an osmotic gradient between the hydrogel and acceptor media. DEX release from both types of IPNs follows zero-order release kinetics (R^2^ > 0.98), with the drug release rate reaching ~86 µg/h, thus providing a dose of ~2 mg per 24 h at a constant release rate. This result demonstrates that the IPN’s composition variation as well as the order of SN synthesis are valuable tools for controlling their capacity as drug delivery systems.

The IPNs composed from PHEMA and PDMAM were found to be non-cytotoxic against T-cell lymphoma cells (HUT-78) and the normal murine fibroblast cell line (CCL-1), which additionally confirms their safe application as dermal drug delivery systems. Future studies on the therapeutic benefits in animal models are expected to further confirm their strong potential in the field. Moreover, the adhesiveness of these novel drug delivery systems will be also studied to further confirm their valuable application for dermal drug delivery. Thus, the study has successfully revealed that IPN hydrogels based on PHEMA and PDMAM are promising candidates for dermal delivery of hydrophobic drugs such as dexamethasone, which is also part of the still not fully revealed potential of general IPNs as delivery systems for controlled drug delivery. The viability of the IPN approach for fine-tuning drug release profiles will be further demonstrated by changing the IPN composition in terms of the novel polymer networks to be included in order to further confirm the opportunities that these polymeric materials could provide for smart drug delivery.

## Figures and Tables

**Table 1:** Descriptions of the straight PDMAM/PHEMA IPNs and both PDMAM SNs used for their preparation.

Designation	C_HEMA_ [mol/L]	$r_{D}^{s}$
**PDMAM SNs**		
**D01**	SN PDMAM with 0.1 mol.% PEGDA	1
**D04**	SN PDMAM with 0.4 mol.% PEGDA	1
**PDMAM/PHEMA IPNs**		
**D** **01H500**	5	0.11
**D** **01H250**	2.5	0.15
**D** **01H125**	1.25	0.26
**D** **04H500**	5	0.13

$r_{D}^{s}$ denotes the weight part of PDMAM in the straight IPNs.

**Table 2:** Descriptions of the reverse PHEMA/PDMAM IPNs and PHEMA SN used for their preparation.

Designation	C_DMAM_ [mol/L]	$r_{D}^{r}$
**PHEMA SN**		
**H1**	SN PHEMA	0
**PHEMA/PDMAM IPNs**		
**H125**	1.25	0.07
**H250**	2.5	0.15
**H500**	5.0	0.31

$r_{D}^{r}$ denotes the weight part of PDMAM in the reverse IPNs.

**Table 3:** Data obtained by applying the main kinetic models on the release profiles of DEX from PHEMA SN, PDMAM SN, reverse PHEMA/PDMAM IPNs, and straight PDMAM/PHEMA IPNs.

PHEMA/PDMAM IPNs									
Model/ Sample Designation	ZO		FO		HM		KP		
	k_0_	R^2^	k_1_	R^2^	k_HM_	R^2^	k_KP_	n	R^2^
**H1**	0.027	0.957	−0.040	0.965	0.097	0.997	0.065	0.671	0.984
**H250**	0.075	0.984	−0.119	0.899	0.270	0.991	0.044	0.996	0.982
**H500**	0.086	0.987	−0.139	0.893	0.310	0.992	0.050	0.981	0.995
**PDMAM/PHEMA IPNs**									
**Model/** **Sample Designation**	**ZO**		**FO**		**HM**		**KP**		
	**k_0_**	**R^2^**	**k_1_**	**R^2^**	**k_HM_**	**R^2^**	**k_KP_**	**n**	**R^2^**
**D01**	0.057	0.981	−0.089	0.922	0.206	0.997	0.032	0.582	0.983
**D01H125**	0.041	0.990	−0.055	0.857	0.146	0.989	0.036	0.753	0.998
**D01H500**	0.038	0.998	−0.059	0.902	0.135	0.970	0.019	0.884	0.987

ZO—zero order; FO—First order; HM—Higuchi model; KP—Korsmeyer–Peppas model.

## Data Availability

The raw/processed data required to reproduce these findings cannot be shared at this time as the data also form part of an ongoing study.

## Supplementary Materials

The following supporting information can be downloaded at: https://www.mdpi.com/article/10.3390/pharmaceutics17010062/s1, Scheme S1. IPN structure where 1st (in red) network does not form covalent bonds with the 2nd network, but both networks interlace and form physical entanglements between themselves. Scheme S2. Structural formulas of HEMA (A), DMAM (B), PEGDA (C), and Dexamethasone (D). Table S1. PDMAM SNs, obtained by using two different monomer (DMAM) and four crosslinking agent (PEGDA) concentrations. Figure S1. Visual appearance of PDMAM SNs with 0.1 mol.% (PDM01) and 0.4 mol.% (PDM04) PEGDA in their dry and swollen states (size not to scale). Figure S2. Visual appearance of PDMAM/PHEMA IPNs, described in Table 1, in their dry and swollen states (size not to scale). Figure S3. Visual appearance of PHEMA/PDMAM IPNs, described in Table 2, in their dry and swollen states (size not to scale). Figure S4. ESR of the reverse PDMAM/PHEMA IPNs as function of the ionic strength of the media. Figure S5. ESR of the straight PDMAM/PHEMA IPNs in water as a function of temperature.

## Appendix A

Photocuring (photopolymerization) is a common process where multifunctional monomers and/or oligomers are converted into a three-dimensional (3D) cross-linked polymeric network through crosslinking chain polymerization, which is initiated by reactive species (free radicals or ions) generated by ultraviolet (UV) irradiation [43]. A factor of significant importance for the reproducibility of the process is the irradiation dose accumulated by the polymerization system. In our study, the experimental setup for the synthesis of IPNs is represented by the UV source and the target—the polymerization system. As a UV source for photopolymerization, we used a MRL-58 Multi-Ray lamp (AnalytikJena, Upland, CA, USA) equipped with a UV-A tube (F8T5/368BL) emitting UV light with 365 nm wavelength at an output power of 1.2 W. The total effective length of the tube from end to end was 26 cm. The polymerization system represents a solution of the monomer (either HEMA or DMAM), crosslinking agent PEGDA, and the UV photoinitiator 1-HCHPK. The solution containing all reagents was casted into a petri dish with a diameter of 5 cm, which was placed at distance of 5 cm below the UV tube. The total dose of UV irradiation for 20 min accumulated by the photopolymerization system was calculated in accordance with the theoretical approach proposed by Keitz [44], which calculates the single-point irradiance E using the following equation:

$$E=\frac{P}{2\pi \mathrm{Ld}}\left[\arcsin\left(\frac{L}{2\sqrt{d^{2}+{\left(\frac{L}{2}\right)}^{2}}}\right)+\arctan\left(\frac{L}{2d}\right)\right]$$

Here:

E is the total irradiance at the measurement point;

P is the total power, emitted by the elongated source equal to 1.2 W;

L is the length of the elongated light source equal to 0.26 m;

d is the perpendicular distance between the light source and the measurement point, which is equal to 0.05 m in this case.

Since the measuring point is not a single point but rather an area with a defined surface, then the average irradiance ($E_{avg})$ is calculated as follows:

$$E_{\mathrm{avg}}=E\times \frac{R^{2}}{d^{2}}$$

where R is the diameter of the circle (the polymerization system’s surface), which in this case is equal to 0.025 m.

The total UV irradiation dose is given as follows:

$$D=E_{\mathrm{avg}}\times t$$

where t is time for irradiation in seconds (1200 s).

By substituting all values used in this study’s experimental setup, the total irradiation dose (D) was calculated to be ~265 J/m^2^.
