# Supplementary material for: Hydrogels of Poly(2-hydroxyethyl methacrylate) and Poly(N,N-dimethylacrylamide) Interpenetrating Polymer Networks as Dermal Delivery Systems for Dexamethasone
Source: Pharmaceutics. 2025 Jan 5;17(1):62. doi: 10.3390/pharmaceutics17010062 (PMC11768119; doi:10.3390/pharmaceutics17010062)
Supplement: Supplementary file 1 [file pharmaceutics-17-00062-s001.zip › pharmaceutics-3356632-supplementary.pdf]

Supplementary Information

to

"Hydrogels of poly(2-hydroxyethyl methacrylate) and poly(N,N-dimethylacrylamide) interpenetrating polymer net-works as dermal delivery systems for dexamethasone"

Marin Simeonov [1,*], Bistra Kostova [2], Rositsa Mihaylova [3] and Elena Vassileva [1]

[1] Laboratory on Structure and Properties of Polymers, Faculty of Chemistry and Pharmacy, University of Sofia, 1, J. Bourchier Blvd., 1164 Sofia, Bulgaria; evassileva@chem.uni-sofia.bg
[2] Department of Pharmaceutical Technology and Biopharmaceutics, Faculty of Pharmacy, Medical University of Sofia, 2, Dunav Str., 1000 Sofia, Bulgaria; bkostova@pharmfac.mu-sofia.bg
[3] Department of Pharmacology, Pharmacotherapy and Toxicology, Faculty of Pharmacy, Medical University of Sofia, 2, Dunav Str., 1000 Sofia, Bulgaria; rmihaylova@pharmfac.mu-sofia.bg
* Correspondence: m.simeonov@chem.uni-sofia.bg

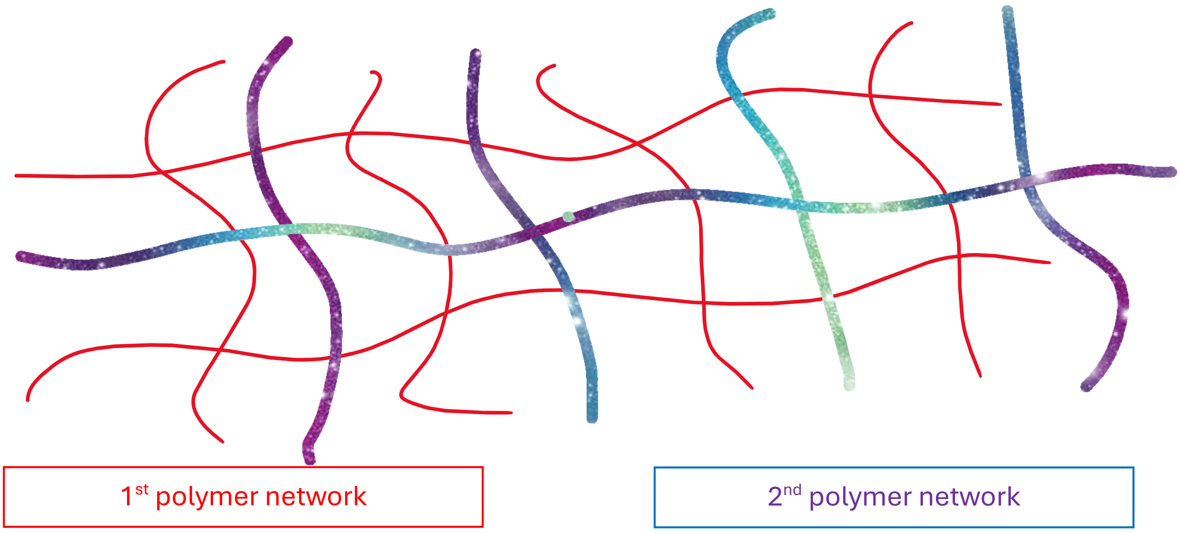

Scheme S1. IPN structure where 1st (in red) network does not form covalent bonds with the 2nd network but both networks interlace and form physical entanglements between themselves.

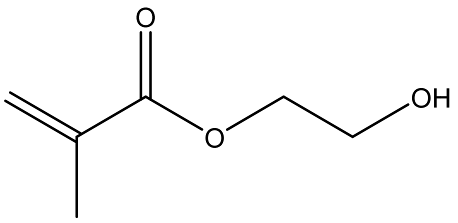

A

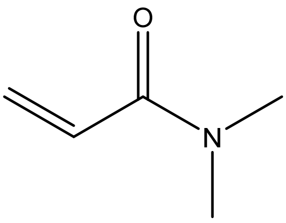

B

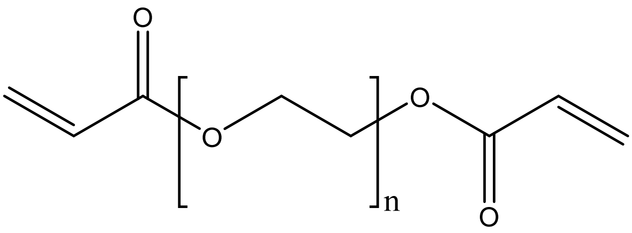

C

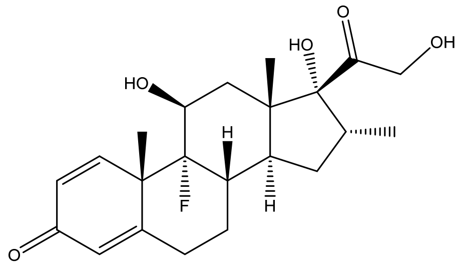

D

Scheme S2. Structural formulas of HEMA (**A**), DMAM (**B**), PEGDA (**C**) and Dexamethasone (DEX) (**D**)

Table S1. PDMAM SNs, obtained by using two different monomer (DMAM) and four crosslinking agent (PEGDA) concentrations.

PDMAM SN designation	C DMAM [mol/l]	PEGDA [mol.%]	HCHPK [mol.%]	Note
**PDM5-4**	5	4	0.1	Brittle
**PDM5-1**	5	1	0.1	Brittle
**PDM5-01**	5	0.1	0.1	Brittle
**PDM3-4**	3	4	0.1	Brittle
**PDM3-1**	3	1	0.1	Brittle
**PDM3-04 (D04)**	3	0.4	0.1	Strong and elastic
**PDM3-01 (D01)**	3	0.1	0.1	Strong and elastic

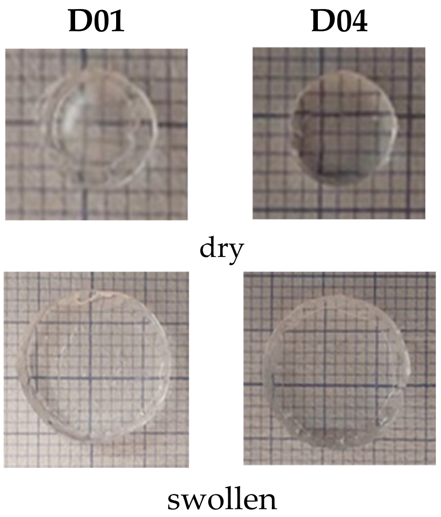

Figure S1. Visual appearance of PDMAM SNs with 0.1 mol.% (PDM01) and 0.4 mol.% (PDM04) PEGDA in their dry and swollen states (size not to scale).

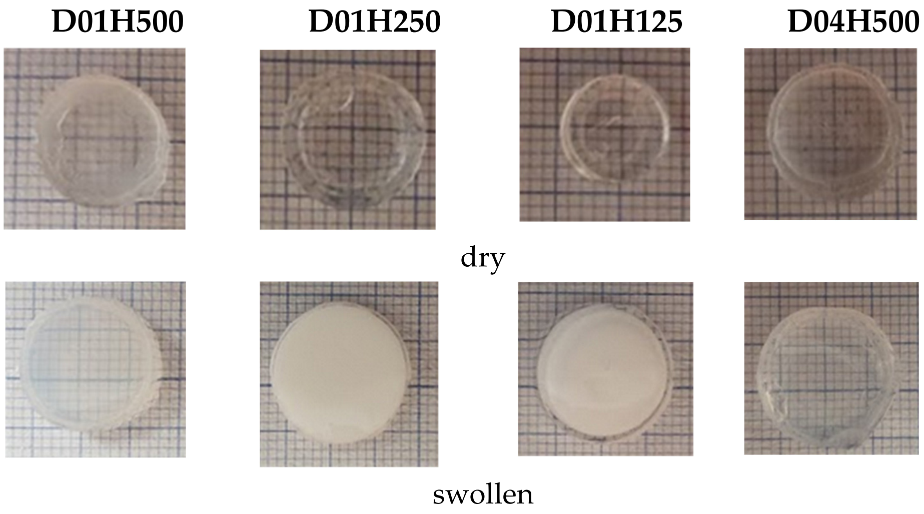

Figure S2. Visual appearance of PDMAM/PHEMA IPNs, described in Table 1, in their dry and swollen states (size not to scale).

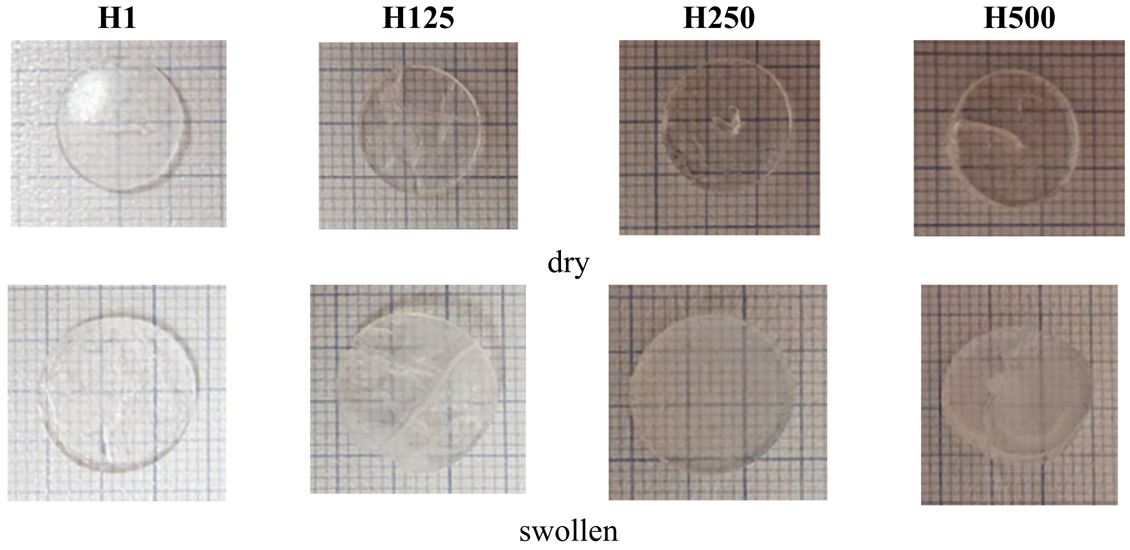

Figure S3. Visual appearance of PHEMA/PDMAM IPNs, described in Table 2, in their dry and swollen states (size not to scale).

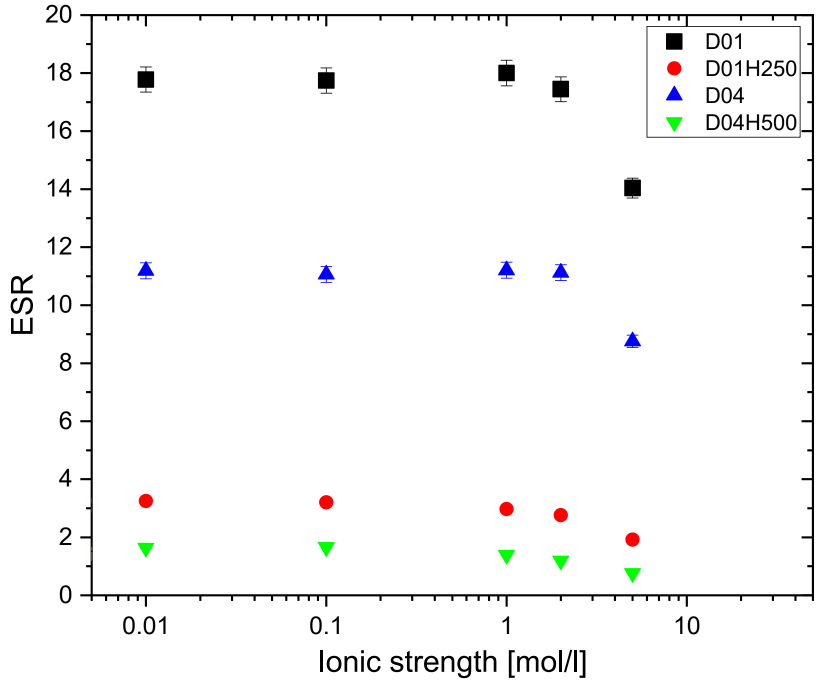

Figure S4. ESR of the reverse PDMAM/PHEMA IPNs as function of the ionic strength of the media.

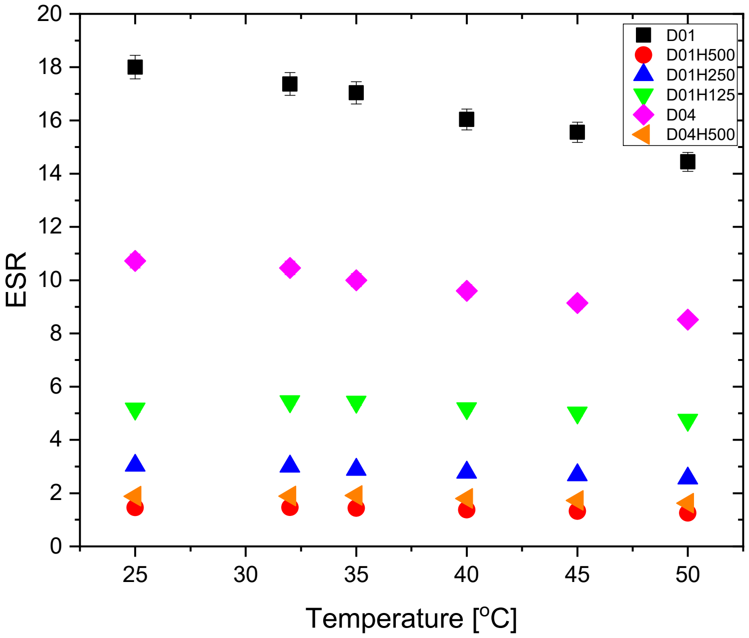

Figure S5. ESR in water of the straight PDMAM/PHEMA IPNs as a function of temperature.
